# Detection and classification of lymphoma from cell-free methylome data

**DOI:** 10.1038/s41698-026-01533-8

**Published:** 2026-06-03

**Authors:** Victoria B. Shelton, Mohamed Alias, Ting Liu, Davidson Zhao, Pamela Alamilla, Althaf Singhawansa, Michael Hong, Yong Zeng, Vanessa Murad, Ibrahim Alrekhais, Ur Metser, David Hodgson, Tomohiro Aoki, Anca Prica, John Kuruvilla, Michael Crump, Bernard Lam, Michael M. Hoffman, Scott V. Bratman, Robert Kridel

**Affiliations:** 1 Princess Margaret Cancer Centre—University Health Network, Toronto, ON Canada; 2 Department of Medical Biophysics, University of Toronto, Toronto, ON Canada; 3 Ontario Institute for Cancer Research, Toronto, ON Canada; 4 Vector Institute, Toronto, ON Canada

**Keywords:** Biomarkers, Cancer, Computational biology and bioinformatics, Oncology

## Abstract

Diagnosing lymphoma traditionally relies on invasive tissue biopsies, which can yield insufficient material for histopathological evaluation and carry a risk of complications. Minimally invasive assessment of cell-free DNA (cfDNA) in plasma offers a promising alternative for lymphoma detection that could aid the rapid evaluation of malignant vs. benign lymphadenopathy. Here, we examine the methylome of plasma samples from 165 lymphoma patients and 47 controls using cell-free methylated DNA immunoprecipitation and high-throughput sequencing (cfMeDIP-seq). Differential methylation analysis of a discovery cohort (142 out of 212 samples) revealed 13,897 hypermethylated genomic regions in lymphoma cases, which were subsequently used for classification using regularized binomial generalized linear models. In a validation cohort (70 samples), we identified lymphomas with an accuracy of 0.89, positive predictive value (PPV) of 0.90 and negative predictive value (NPV) of 0.87. cfDNA methylation scores were significantly associated with orthogonal measures of cfDNA tumor burden, stage, and clinical outcomes. Our results highlight the feasibility of cfDNA methylation profiling as a sensitive and minimally invasive method for detecting lymphoma.

## Introduction

Lymphomas represent a heterogeneous group of diseases that currently require invasive histological diagnosis to inform treatment decisions^1^. The standard diagnostic approach relies on excisional or needle core biopsies, followed by histopathological assessment. Not only are these tissue biopsies painful or uncomfortable for patients, but due to the limited tissue sampling, they may not fully capture the underlying heterogeneity of lymphoma. This holds especially true for needle core biopsies, which are increasingly used as a less invasive diagnostic method but may provide insufficient material for comprehensive analysis in up to 39% of patients^2–6^. This necessitates additional biopsies and causes treatment delays and patient uncertainty.

Blood-based biomarkers offer a less invasive alternative to tissue biopsies, minimizing patient discomfort and reducing the risk of complications. Laboratory analysis of blood samples may even eliminate the need for surgical interventions in some cases. Previous investigations show the potential of minimally invasive liquid biopsies from blood samples in a wide variety of cancer types^7^. Liquid biopsies can detect and classify malignancies, dynamically assess tumor burden during treatment, and evaluate the depth of response after completion of cancer-directed therapy^8,9^.

In the lymphoma context, genomic analysis of cell-free DNA (cfDNA) provides a window into the genetic landscape of lymphomas, enabling the detection and monitoring of lymphoma-associated mutations^10–12^. However, gene mutations alone may not capture the full range of molecular aberrations or explain the extent of inter-patient variability observed in the clinical setting. cfDNA methylation profiling has emerged as a promising strategy, offering a complementary approach by quantifying epigenetic alterations^13^. This technique provides potentially thousands of methylation data points per sample, offering a rich layer of information that may further refine the understanding of lymphoma biology and improve diagnostic accuracy. The effectiveness of this approach stems from the unique DNA methylation signatures characteristic of the malignant state^14^.

Here, we leveraged cell-free Methylated DNA immunoprecipitation and high-throughput sequencing (cfMeDIP–seq), an enrichment-based method for profiling aberrant methylation patterns across the genome, to non-invasively detect and classify lymphoma^13,15^. We focused on diffuse large B-cell lymphoma (DLBCL) and follicular lymphoma (FL), the most common non-Hodgkin lymphoma subtypes, as well as classical Hodgkin lymphoma (HL), which together represent approximately 60% of all lymphoma diagnoses. Using cfMeDIP-seq, we were able to non-invasively identify the methylation fingerprints of these lymphoma subtypes, demonstrating feasibility of lymphoma detection from plasma and showing correlations with orthogonal measures of tumor burden. Our results highlight the promise of cfMeDIP-seq to improve diagnostic workflows for lymphoma patients.

## Results

### Generation of cell-free methylation profiles from lymphoma patients

**Fig. 1:**
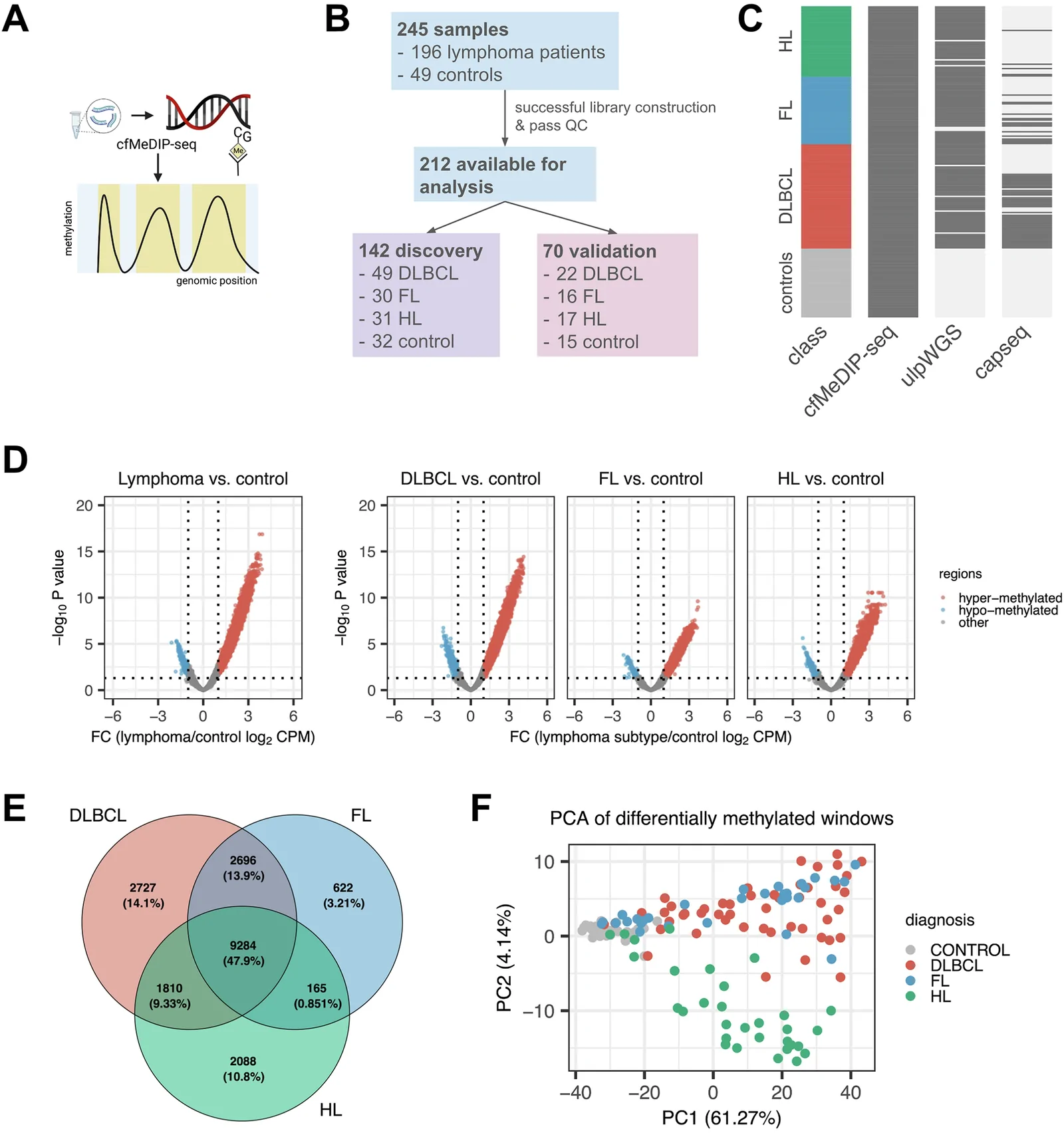
Overview of Study Design and Differential Methylation Analysis. **A** Schematic representation of cfMeDIP-seq. **B** Summary of study cohorts with sample counts stratified by diagnosis. **C** Distribution of samples profiled by cfMeDIP-seq, indicating overlap with ultra-low-pass whole genome sequencing (ulpWGS) and targeted capture sequencing (capseq). **D** Volcano plots illustrating differentially methylated genomic regions across diagnostic contrasts. **E** Venn diagram showing the overlap of differentially methylated regions among contrasts. **F** Principal component analysis (PCA) based on the top 1000 differentially methylated regions, with lymphoma subtypes indicated by color.

A total of 245 plasma samples, from 196 lymphoma patients and 49 controls, were subjected to cfDNA extraction followed by cfMeDIP-seq (Fig. 1A–B). Library construction failed for 7 samples due to insufficient yield after cfDNA extraction. Setting a quality filter requiring at least 10 million usable reads resulted in 212 samples meeting quality control criteria: 165 lymphoma and 47 controls (Fig. 1B). The lymphoma samples comprised 71 DLBCL, 46 FL and 48 HL cases. Patient characteristics are summarized in Table 1. The majority of samples (84.2%) were collected from the time point of diagnosis. Advanced and limited stage disease was noted in 59.4% and 40.6% of patients, respectively. The cohort was split into discovery (*n* = 142, 67.0%) and validation cohorts (*n* = 70, 33.0%) to facilitate model building and validation. Clinical indices were evenly distributed between the discovery and validation cohorts (Table 1).

**Table 1:** Patient characteristics

		Combined (*n* = 212)	Discovery cohort (*n* = 142)	Validation cohort (*n* = 70)	*p*
Age	≤ 60	130 (61.3%)	86 (60.6%)	44 (62.9%)	
	> 60	82 (38.7%)	56 (39.4%)	26 (37.1%)	0.863
Sex	M	108 (50.9%)	67 (47.2%)	41 (58.6%)	
	F	104 (49.1%)	75 (52.8%)	29 (41.4%)	0.157
Diagnosis	DLBCL	71 (33.5%)	49 (34.5%)	22 (31.4%)	
	FL	46 (21.7%)	30 (21.1%)	16 (22.9%)	
	HL	48 (22.6%)	31 (21.8%)	17 (24.3%)	
	Control	47 (22.2%)	32 (22.5%)	15 (21.4%)	0.951
Type	Newly diagnosed	139 (84.2%)	92 (83.6%)	47 (85.5%)	
	First relapse	26 (15.8%)	18 (16.4%)	8 (14.5%)	0.940
	NA (controls)	47	32	15	
Stage	I	21 (12.7%)	15 (13.6%)	6 (10.9%)	
	II	46 (27.9%)	31 (28.2%)	15 (27.3%)	
	III	31 (18.8%)	21 (19.1%)	10 (18.2%)	
	IV	67 (40.6%)	43 (39.1%)	24 (43.6%)	0.934
	NA (controls)	47	32	15	

For samples passing quality control, the median number of raw and usable reads after deduplication was 84.5 million (interquartile range (IQR) 70.7–100.1) and 38.2 million (IQR 24.9–48.1), respectively. The median of the per-sample fragment size modes was 166 bp. The median relative methylation enrichment and the median G + C normalized methylation were 3.43 (IQR 3.14–3.69) and 2.08 (IQR 1.95–2.24), respectively, showing good methylation enrichment, even after adjusting for GC content. The data were therefore deemed to be of high quality and suitable for downstream analysis. To provide orthogonal measurements of tumor burden from cfDNA, we performed ultra-low pass whole genome sequencing (ulpWGS) of cfDNA in 155 cases (93.9%) and hybrid capture-based sequencing (capseq) in 65 cases (39.4%) out of the 165 lymphoma samples that passed cfMeDIP-seq quality control (Fig. 1C).

### Delineation of lymphoma samples from healthy controls

To gain insight into lymphoma-associated methylation of cfDNA, we performed differential methylation analysis, comparing lymphoma cases to controls (Fig. 1D). This analysis revealed a strong signal of hypermethylation, with 13,897 hypermethylated and 496 hypomethylated regions, using an absolute log_2_ fold-change threshold of 1 and an adjusted p-value cut-off of 0.05. To explore methylation differences across specific lymphoma subtypes, we fitted a linear model with three comparisons: DLBCL vs. controls, FL vs. controls, and HL vs. controls. Of all differentially methylated regions, 47.7% were shared across all three contrasts, indicating the presence of common methylation patterns (Fig. 1E). A signal of predominant hypermethylation was seen for all lymphoma subtypes, with 15,422, 12,392, and 12,724 hypermethylated regions for DLBCL, FL, and HL, respectively. Consistent with DLBCL and FL being both derived from germinal centre B cells, they shared a higher number of hypermethylated regions when comparing either to HL. FL had the lowest number of uniquely hypermethylated regions. Principal component analysis of the top 1000 differentially methylated regions showed distinct, though imperfect, separation between lymphoma and control cases along the first principal component, which accounted for 61.3% of the variation (Fig. 1F). DLBCL and FL cases did not exhibit clear separation, whereas the majority of HL cases clustered distinctly along the second principal component.

### Validation of lymphoma-specific methylation in plasma using tissue data and fragment analysis

**Fig. 2:**
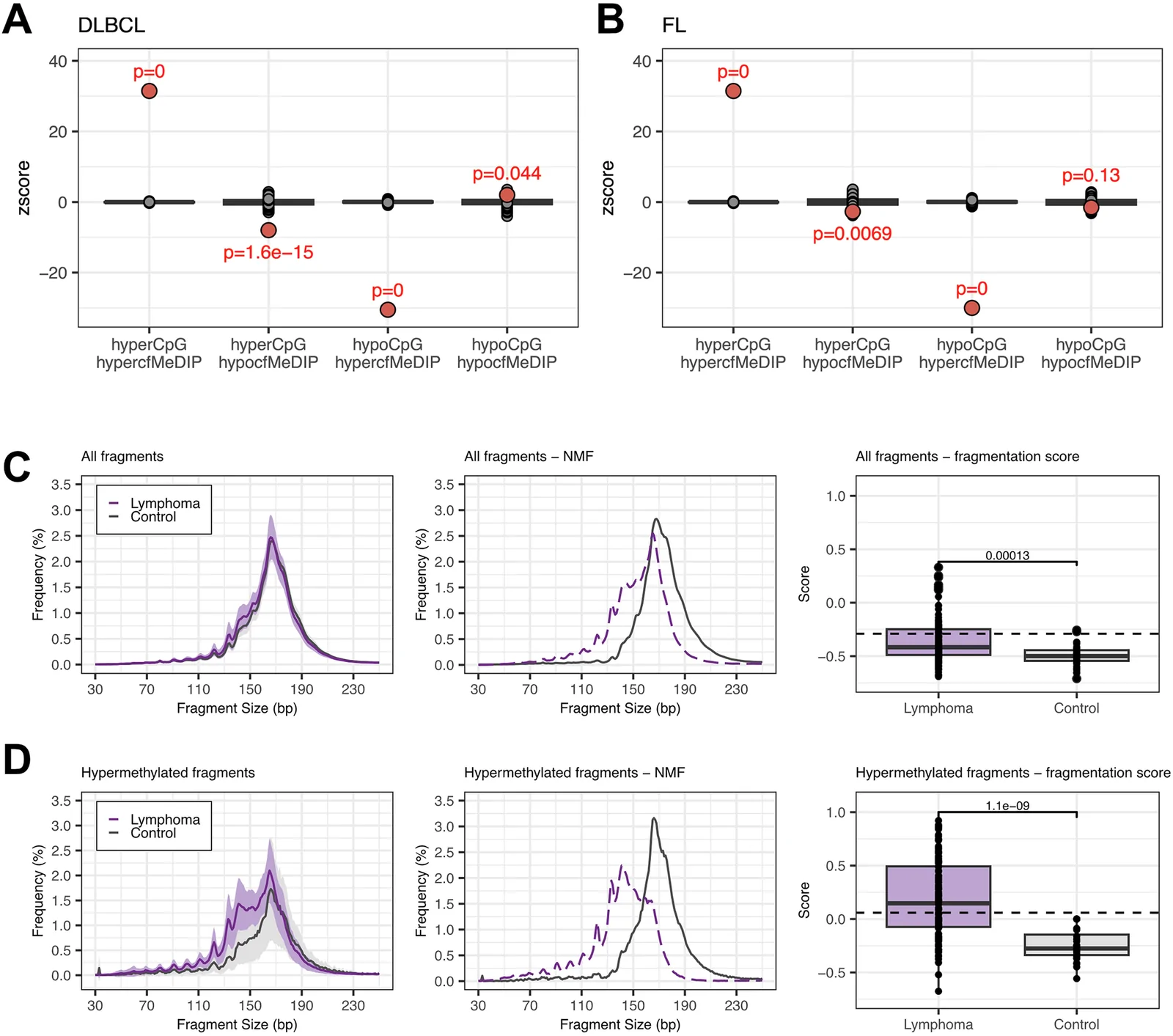
Validation of the Tumor-Specificity of the cfMeDIP-seq Methylation Signals and Assessment of Fragment Size Distributions. **A** Permutation testing demonstrating the enrichment of differentially methylated CpGs—identified in an orthogonal tissue-based methylation dataset (Illumina EPIC array)—within cfMeDIP-seq-defined differentially methylated regions. Results are shown separately for diffuse large B-cell lymphoma (DLBCL, left) and follicular lymphoma (FL, right). **B** Analysis of cfMeDIP-seq fragment size characteristics. Histograms display fragment size distributions (left column), unsupervised rank-2 non-negative matrix factorization (NMF) component weights (middle column), and fragmentation scores (right column), for all reads **C** and **D** reads overlapping hypermethylated regions.

To verify that the methylation changes observed in plasma were specific to lymphoma and not attributable to technical artifacts or noise, we utilized in-house tissue-based methylation data generated using Infinium MethylationEPIC BeadChips on an orthogonal dataset of DLBCL (*n* = 157) and FL (*n* = 338) cases. Using permutation testing, we identified a strong enrichment of hypermethylated CpGs within hypermethylated regions in plasma for both DLBCL and FL (Fig. 2A-B). In DLBCL, hypomethylated CpGs were weakly enriched within hypomethylated regions—a pattern not observed in FL. However, consistent with expectations, hypomethylated CpGs were negatively associated with hypermethylated regions in both DLBCL and FL. These observations suggest that the differential methylation signal observed in plasma accurately reflects lymphoma-specific methylation patterns in tumor tissue.

Next, we examined fragment length distributions in cfMeDIP-seq libraries from lymphoma cases and controls (Fig. 2C). Fragments from lymphoma cases had a tendency toward shorter fragment sizes, a pattern further supported by unsupervised rank-2 non-negative matrix factorization (NMF), which separated samples into components reflecting differential fragment length distributions consistent with tumor- and non-tumor-derived cfDNA. This trend was further corroborated by fragmentation score analysis, with scores calculated using the method described by Vessies et al.^16^, and became more pronounced when focusing on fragments mapping exclusively to hypermethylated regions (Fig. 2D). As tumor-derived cfDNA has been shown to exhibit altered fragmentation profiles, with a higher proportion of short fragments^17^, these findings indicate that hypermethylated reads captured through cfMeDIP-seq are reflective of tumor-specific methylation changes.

### Genomic annotation of differentially methylated regions

**Fig. 3:**
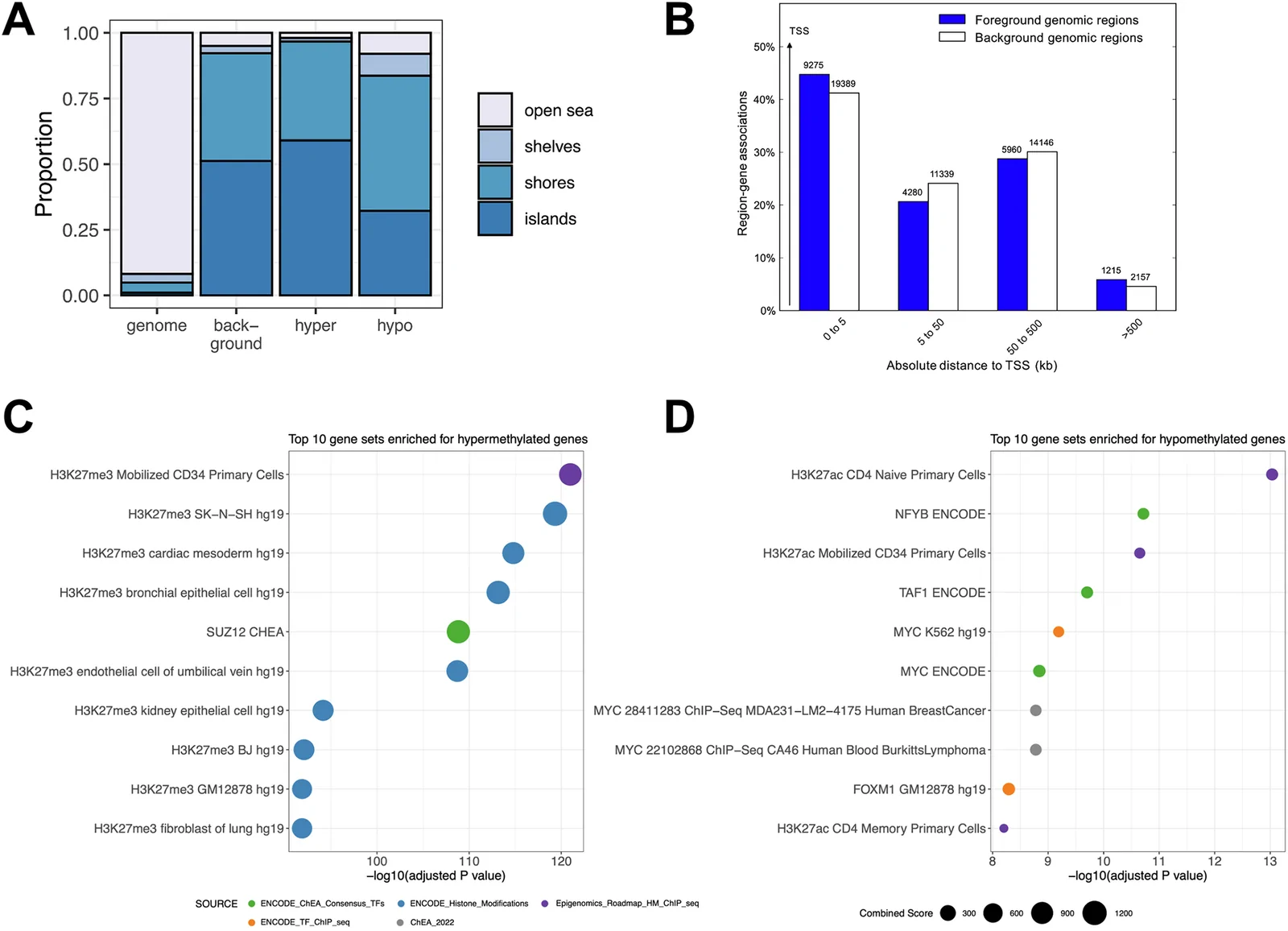
Genomic and Epigenomic Context and Annotation of Differentially Methylated Regions in Lymphoma. **A** Stacked bar plot showing the distribution of differentially methylated regions across CpG features, including islands, shores, shelves, and open sea regions. **B** Barplot showing the number of differentially methylated regions in relation to the distance to the nearest transcription start site (TSS). **C**, **D** Dot plots illustrating the top ten gene sets enriched among genes with hypermethylated (**C**) and hypomethylated (**D**) promoter-proximal regions (within 1 kb of the TSS), based on gene set enrichment analysis.

Given that the cfMeDIP-seq technique selectively enriches for methylated CpG regions, we anticipated a strong enrichment of CpG islands and shores within the filtered genomic regions (referred to as the “background”) in our dataset. In lymphoma cases, hypermethylated regions exhibited increased enrichment of CpG islands and shores relative to background regions. (96.7% vs. 92.2%, *P* < 0.001). Conversely, the hypomethylated regions were relatively depleted of CpG islands and shores (83.7% vs. 92.2%, *P* < 0.001) (Fig. 3A). Both hypermethylated and hypomethylated regions were enriched near transcription start sites (TSS), with 44.7% (Fig. 3B) and 39.3% of region-gene associations, respectively, located within 0–5 kbp of the TSS. Gene annotations from hypermethylated promoter regions (within 1 kbp of the transcription start site) showed strong enrichment for transcriptional regulation, developmental pathways, and synaptic function and cell communication (Supplementary Data 1), suggesting regulation of broad transcriptional networks through DNA methylation. We next incorporated epigenetic annotations and found that hypermethylated genes were significantly enriched for those marked by the repressive histone modification H3K27me3 across diverse cellular contexts (Fig. 3C). In contrast, hypomethylated genes showed enrichment for gene sets associated with H3K27ac, an activating histone modification, as well as for the MYC transcription factor (Fig. 3D). These findings are consistent with a previous study that reported an over-representation of genes repressed in stem cells by Polycomb Repressive Complex 2 (PRC2) among hypermethylated genes in FL^18^. Taken together, these results demonstrate that cfMeDIP-seq effectively captures proximal regulatory regions, with concordant enrichment of repressive histone methylation and DNA hypermethylation.

### Classification of lymphoma versus controls

**Fig. 4:**
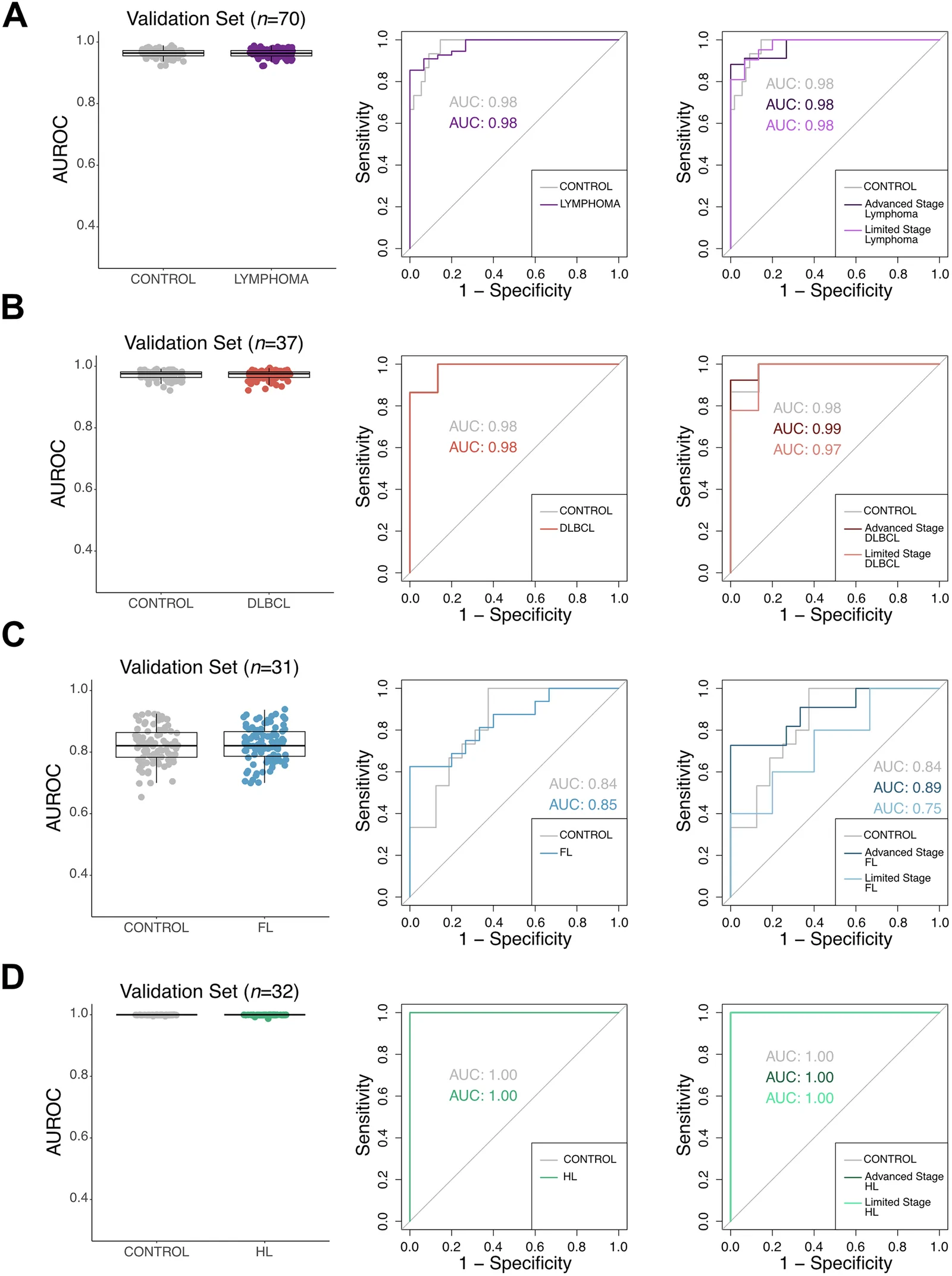
Performance of binary machine learning classification models in the validation cohort. Classification tasks shown are: **A** Lymphoma vs. control (*n* = 70), **B** DLBCL vs. control (*n* = 37), **C** FL vs. control (*n* = 31), and **D** HL vs. control (*n* = 32). For each task, the left plot shows the distribution of area under the received operating characteristic curve (AUROC) across 100 independent model iterations; the middle plot shows the receiver operating characteristic curve (ROC); and the right plot shows the stage-stratified ROC curves (limited = Ann Arbor I–II; advanced = III–IV; with line colors distinguish limited vs advanced stage as shown in the plots).

Building on the results of our methylated region analysis, we applied machine learning approaches to classify samples based on cfMeDIP-seq profiles. As a first step, we assessed whether our classification model could reliably distinguish between healthy controls and lymphoma samples (Fig. 4A). This two-class classification model achieved an accuracy of 0.89 for lymphoma detection in the validation cohort, with a positive predictive value (PPV) of 0.9, a negative predictive value (NPV) of 0.87, a sensitivity of 0.97 (Table 2) and an area under the curve (AUC) of 0.98. While sensitivity was high, specificity was more modest (58%), reflecting the inherent trade-off between these metrics. Next, we constructed binary classification models to distinguish between healthy controls and each diagnosis class under investigation. The DLBCL vs. control classification model yielded an AUC of 0.98 and an accuracy of 0.89 (PPV = 0.95, NPV = 0.83) (Fig. 4B, Supplementary Data 2). The FL vs. control classifier produced an AUC of 0.85 and accuracy of 0.76 (PPV = 0.89, NPV = 0.69) (Fig. 4C, Supplementary Data 2). Finally, the HL vs. control model achieved an AUC of 1.00 and an accuracy of 0.99 (PPV = 1, NPV = 0.98) (Fig. 4D, Supplementary Data 2).

**Table 2:** Performance metrics of two-class models in validation cohort

	Controls vs Lymphomas			
Performance Metrics^a^	Control Classification (*n* = 15)	Lymphoma Classification (All Stages) (*n* = 55)	Lymphoma Classification (Stage 1 & 2) (*n* = 21)	Lymphoma Classification (Stage 3 & 4) (*n* = 34)
Accuracy	0.82	0.89	0.82	0.85
Kappa	0.49	0.62	0.60	0.59
Sensitivity	0.58	0.97	0.99	0.96
Specificity	0.88	0.58	0.58	0.58
Positive Predictive Value	0.72	0.90	0.77	0.84
Negative Predictive Value	0.89	0.87	0.98	0.89
Precision	0.72	0.90	0.77	0.84
Recall	0.58	0.97	0.99	0.96
F1	0.59	0.93	0.87	0.90

^a^100-iteration mean values.

To further evaluate the classification performance of the binary models, we assessed their ability to discriminate between limited and advanced-stage samples. The lymphoma vs. control classification model achieved an AUC score of 0.98 for advanced-stage lymphoma samples and 0.98 for limited-stage lymphoma (Fig. 4A). The DLBCL, FL, and HL vs. control models achieved AUCs of 0.99 and 0.97, 0.89 and 0.75, and 1 and 1 for advanced- and limited-stage samples, respectively (Fig. 4B–D). Overall, the binary models demonstrated robust performance in classifying advanced-stage samples, with most models also achieving high classification accuracy for limited-stage samples.

### Classification of lymphoma subtypes

**Fig. 5:**
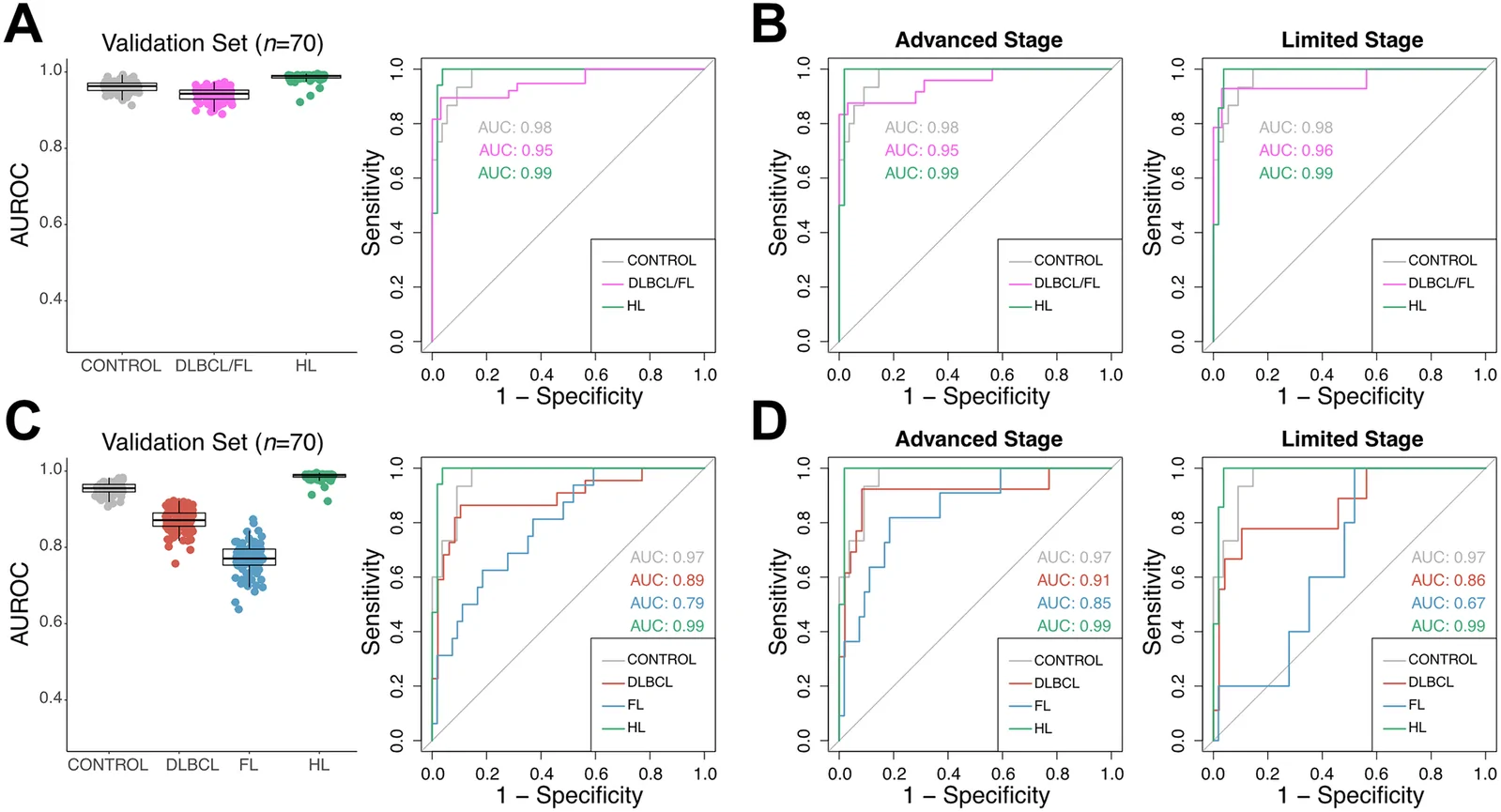
Performance of Multi-Class Machine Learning Classifiers in the Validation Cohort. **A**, **B** Area under the receiver operating characteristic (AUROC) scores from 100 iterations of classification models, comparing performance across diagnostic groups for two classification schemes: (**A**) DLBCL/FL vs. HL vs. control, and (**B**) DLBCL vs. FL vs. HL vs. control. receiver operating characteristic (ROC) curves showing model performance stratified by disease stage (limited vs. advanced) for the same classification schemes: **C** DLBCL/FL vs. HL vs. control, and **D** DLBCL vs. FL vs. HL vs. control.

We next evaluated the performance of a three-class classification model trained to differentiate among healthy controls, a combined group of DLBCL and FL samples, and HL samples. This model achieved high classification metrics across all categories, with the HL group showing the highest classification confidence. For HL classification, the model achieved an AUC of 0.99 (Fig. 5A), an accuracy of 0.89, a PPV of 0.91 and a NPV of 0.88 in the validation cohort (Supplementary Data 3). For the DLBCL/FL group, the model yielded an AUC of 0.95 and an accuracy of 0.87, with PPV and NPV values of 0.93 and 0.82, respectively. The model’s AUC for control classification was 0.98, with an accuracy of 0.81, a PPV of 0.69, and an NPV of 0.89. Additionally, we evaluated the model’s ability to discriminate between limited and advanced-stage samples, noting similar AUC values for advanced-stage HL and DLBCL/FL samples compared to limited-stage cases (Fig. 5B).

Finally, we developed a classification model to differentiate between all four diagnostic classes (controls, HL, DLBCL, and FL). In this model, the classification performance was again the highest for HL (AUC 0.99), followed by DLBCL (AUC 0.89) and FL (AUC 0.79) (Fig. 5C, Supplementary Data 4). Sensitivity for FL was low (10%), reflecting the biological similarity between FL and DLBCL. In the three-class model, we grouped these two subtypes into a single DLBCL/FL subtype resulting in substantially increased sensitivity (0.82). The AUC scores for advanced-stage samples were higher than those for limited-stage samples (Fig. 5D). Taken together, these results indicate that the multi-class models classified HL with the highest accuracy, whereas FL proved more challenging to distinguish from DLBCL.

### Investigation of sources of classification uncertainty

While the accuracy was high in our binary classification models, with 98.2%, 86.4%, and 100% of lymphoma, DLBCL, and HL cases correctly classified, respectively, we observed a higher rate of incorrect classifications when comparing FL to controls, with only 56.3% of FL cases correctly classified. We hypothesized that misclassification could be driven by low tumor burden. We first measured the bulk of viable tumor on standard-of-care FDG-PET-CT (^18^F-fluorodeoxyglucose positron emission tomography–computed tomography) scans. Among 59 DLBCL cases with available baseline PET-CT data, those with low metabolic tumor volume (MTV) were more likely to be misclassified and had significantly higher control classification scores in the four-class model (Wilcoxon *p* = 6.5 × 10^-6^) (Supplementary Fig. 1).

Next, we assessed whether tumor fraction within cfDNA, as measured via ulpWGS, influenced classification accuracy. Among validation cohort samples with ulpWGS data (7 Control, 21 DLBCL, 16 FL, 17 HL), classification scores were significantly correlated with tumor fraction concentration (Spearman *p* = 2 × 10^−12^; Supplementary Fig. 2A), a trend consistent across all binary models (Supplementary Fig. 2B–F). Finally, we explored mutation profiles in DLBCL samples from both discovery and validation cohorts with matched cfDNA capseq data (Supplementary Fig. 3A). DLBCL samples more likely to be misclassified as FL had an enrichment of germinal-centre lymphoma type mutations in *BCL2*, *CREBBP* and *EZH2* and had infrequent mutations associated with activated B-cell-like DLBCL in *PRDM1*, *CCND3*, *BTG1* and *TBL1XR1* (Supplementary Fig. 3B). These observations suggest that tumor burden and underlying disease biology can both contribute to classification variability.

### Association of cfDNA methylation scores with tumor burden

**Fig. 6:**
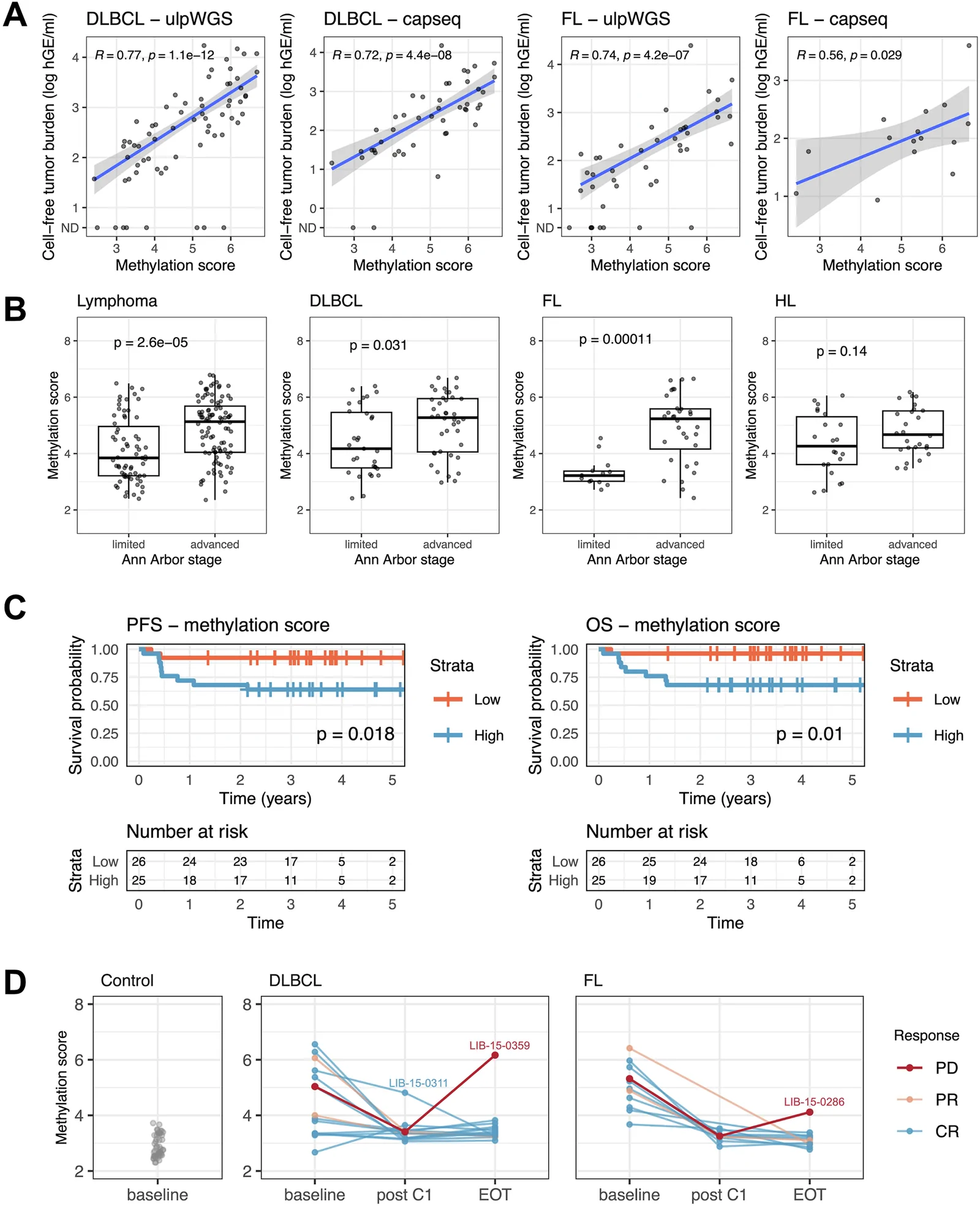
Association of cell-free methylation scores with tumor burden, disease stage and patient outcomes. **A** Scatter plots illustrating the correlation between cell-free methylation scores (x-axes) and circulating tumor burden (y-axes, log hGE/mL), as estimated by either ultra-low-pass whole genome sequencing (ulpWGS) or targeted capture sequencing (capseq), in DLBCL and FL samples. **B** Box plots comparing cell-free methylation scores across disease stages, shown for all lymphoma cases combined, as well as stratified by DLBCL, FL, and HL subtypes. **C** Kaplan–Meier survival curves showing progression-free survival (PFS, left) and overall survival (OS, right) in DLBCL patients, stratified by methylation score groups (low vs. high, dichotomized at the median). **D** Methylation scores within lymphoma-associated hypermethylated regions hypermethylated: Scores are shown at baseline in controls (left panel) and across three time points—baseline, post cycle 1 (C1) and at end-of-treatment (EOT)—in patients with DLBCL and FL treated with immunochemotherapy.

For each individual sample, we calculated lymphoma subtype-specific methylation scores, defined as the mean normalized methylation values of hypermethylated genomic regions (identified using a log_2_ fold-change threshold of 1 and an adjusted p-value cutoff of 0.05). For a subset of cases, we generated orthogonal measurements of tumor burden within cfDNA, employing either ulpWGS (*n* = 67 DLBCL, *n* = 43 FL, *n* = 45 HL) or capseq (*n* = 45 DLBCL, *n* = 15 FL, *n* = 5 HL). We observed significant correlations between cfDNA methylation scores and the tumor burden within cfDNA estimated by ulpWGS (*R* = 0.77, *p* = 1.1 × 10^−12^) or capseq (*R* = 0.72, *p* = 4.4 × 10^−8^) for DLBCL, with similar trends noted for FL (*R* = 0.74, *p* = 4.2 × 10^−7^ for ulpWGS and *R* = 0.56, *p* = 0.029 for capseq) (Fig. 6A). Unexpectedly, we found no significant correlation between cfDNA methylation scores and tumor fraction within cfDNA as estimated by ulpWGS in HL (*R* = 0.19, *p* = 0.22).

Next, we investigated the association between cfDNA methylation scores and clinical indices of tumor burden and prognosis, including Ann Arbor stage, serum LDH, prognostic indices and MTV. We found significant differences between limited and advanced stages for lymphoma cases (*p* = 2.6 × 10^-5^), with differences particularly marked for FL (*p* = 1.1 × 10^-4^) and less so for DLBCL (*p* = 0.031) and HL (*p* = 0.14) (Fig. 6B). Methylation scores were significantly associated with LDH levels in both DLBCL (*R* = 0.53, *p* = 2.0 × 10⁻⁶) and FL (*R* = 0.50, *p* = 3.7 × 10⁻⁴) (Supplementary Fig. 4A). Additionally, higher methylation scores correlated with increased risk based on the International Prognostic Index (IPI) in DLBCL (*p* = 0.0077) and the Follicular Lymphoma International Prognostic Index (FLIPI) in FL (*p* = 9.1 × 10⁻⁸) (Supplementary Fig. 4B). Lastly, methylation levels correlated with MTV in DLBCL (*R* = 0.65, p = 2.2 × 10⁻⁸) and FL (*R* = 0.66, p = 0.014) (Supplementary Fig. 4C).

### Association of cfDNA methylation scores with patient outcomes

Finally, we assessed the prognostic value of cfDNA methylation scores at baseline and during treatment. First, we performed Kaplan-Meier analysis in newly diagnosed DLBCL patients treated with curative intent immunochemotherapy. Among this subset of 51 patients, 11 progression-free survival (PFS) events occurred. Methylation scores were dichotomized at the median, showing that high scores were significantly associated with inferior PFS (log-rank *p* = 0.018) and overall survival (OS) (log-rank *p* = 0.01) (Fig. 6C). To assess independence from clinical risk factors, we used a stratified Cox model using IPI as the stratification variable. This model showed a hazard ratio for PFS of 4.75 (95% confidence interval 1.01–22.4, *p* = 0.049), indicating that cell-free methylation had independent prognostic value. We next evaluated whether the prognostic association of cfDNA methylation scores was retained after accounting for disease stage. In stage-stratified Kaplan–Meier analyses, high methylation scores were associated with inferior overall survival among patients with advanced-stage disease (log-rank *p* = 0.014), with a trend toward shorter progression-free survival (*p* = 0.065) (Supplementary Fig. 5A-B). In contrast, no significant differences in progression-free or overall survival were observed among patients with limited-stage disease (PFS *p* = 0.31; OS *p* = 0.73), noting the small number of patients and events in this subgroup (Supplementary Fig. 5C-D).

Serial plasma samples (post-cycle 1 and end-of-treatment (EOT)) were available for 15 DLBCL and 12 FL patients, with post-cycle 1 samples missing in two FL cases (Fig. 6D). These were analyzed in relation to response assessed by functional and/or cross-sectional imaging (EOT PET-CT scans were available for all DLBCL patients and for 10 FL patients). One DLBCL patient (LIB-15-0359) showed progressive disease on EOT PET-CT following an initial radiologic response, accompanied by a marked increase in cfDNA methylation score at EOT. Similarly, one FL patient (LIB-15-0286) was found to have histologic transformation at EOT, which was also associated with a rise in methylation score after an initial decline. Another DLBCL patient (LIB-15-0311) demonstrated a slow decline in methylation score and experienced early progression two months after achieving a complete metabolic response on EOT PET-CT. Three additional DLBCL patients experienced relapse and one additional FL patient experienced transformation, despite achieving a complete metabolic response and having low methylation scores at EOT. In these latter patients, the median time from EOT to progression was 8 months (range: 5–25 months) for DLBCL and 5 months in the FL case. In contrast, two DLBCL and two FL patients had a partial response on EOT PET-CT and have remained progression-free. These patients had methylation scores at EOT comparable to those seen in healthy controls. Together, these results demonstrate that cell-free methylation scores reflect disease burden and clinical risk in both DLBCL and FL.

## Discussion

In this study, we applied cfMeDIP-seq to a large cohort of plasma samples from lymphoma patients and controls. Our analysis demonstrated delineation of lymphoma samples from controls across all three lymphoma subtypes examined. Notably, the performance metrics were superior in advanced-stage patients compared to those with limited-stage disease. Additionally, cfDNA methylation scores showed strong correlations with other measures of tumor burden within cfDNA, as well as Ann Arbor stage, prognostic indices, and patient outcomes. Prognostic separation was most evident in advanced-stage disease, where tumor burden was higher, and event rates were sufficient to assess outcome differences.

Our findings underscore that cfDNA methylation analysis represents an emerging strategy for non-invasive lymphoma detection. In a study focused on multi-cancer early detection, whole-genome DNA methylation profiling demonstrated superior accuracy in identifying the cancer signal origin compared to other genomic approaches^19^. Targeted methylation profiling is also capable of detecting lymphoma in individuals without signs or symptoms of cancer^20^. Witzig et al. demonstrated that just 16 methylation markers could identify distinct lymphoma subtypes, although marginal zone lymphoma and T-cell lymphoma were more challenging to identify compared to HL, FL, DLBCL, or mantle cell lymphoma^21^. Zhang et al. integrated targeted cell-free methylation profiling with other genetic and epigenetic features from cfDNA to achieve high classification accuracy for DLBCL^22^. In addition to 5-methylcytosine, profiling 5-hydroxymethylcytosine from cfDNA has also shown promise for lymphoma detection and subtype classification^23–26^. Together, these studies highlight the growing potential of cfDNA methylation profiling as a powerful and versatile approach for both detecting lymphoma and distinguishing between its diverse subtypes.

To the best of our knowledge, this study is the first to apply cfMeDIP-seq to samples from lymphoma patients. cfMeDIP-seq is an enrichment-based technique that enables comprehensive profiling of global DNA methylation patterns. We demonstrate that this method achieves high accuracy for the non-invasive detection of lymphoma, with high PPV and NPV. These findings are particularly relevant in the context of rapid diagnostic clinics for lymphoma, where disease prevalence may closely mirror that of our study cohort^6^. While multi-cancer early detection tests may enable early identification of lymphoma^20^, the clinical utility of early detection in lymphoma remains a subject of debate. Indeed, patients with advanced-stage lymphoma may experience favorable outcomes, and a recent study found that patients with incidentally detected DLBCL had outcomes comparable to those diagnosed based on clinical symptoms^27^. Nevertheless, the diagnostic pathway for lymphoma is often lengthy and complex, frequently requiring multiple biopsies before a definitive diagnosis is achieved. A blood-based assay such as cfMeDIP-seq could offer meaningful diagnostic utility. Furthermore, its broad methylation coverage may extend its application to monitoring treatment response and assessing minimal residual disease.

Our study has several limitations. While we included the most common lymphoma subtypes—representing the majority of diagnoses—the utility of cfMeDIP-seq in rarer subtypes, including T-cell malignancies, remains to be established. Furthermore, our classification approach was based solely on methylation features. Integrating additional biomarkers, such as somatic mutations, may further enhance the accuracy and robustness of lymphoma detection. Our model did not reliably distinguish between DLBCL and FL, indicating that the cell-free methylation profiles of DLBCL—particularly those of the GCB subtype—share similarities with those of FL. This overlap suggests that, without additional classifier training, cfMeDIP-seq may have limited resolution for differentiating between aggressive and indolent lymphoma subtypes. Further, given lower performance at low tumor burden, we do not position cfMeDIP-seq as a population-level early detection test; rather, its intended use is as a minimally invasive diagnostic adjunct to help delineate lymphoma from benign lymphadenopathy when a biopsy is challenging or repeatedly non-diagnostic. Expansion of training/validation to include additional lymphoma entities (e.g. other B-cell entities and T-cell lymphomas), non-lymphoid malignancies, and benign conditions will be required to improve generalizability. Our analysis of serial time points was exploratory and not intended to establish cfMeDIP-seq as a clinical minimal residual disease detection assay. Finally, the cfDNA methylation score used in this study was designed for lymphoma detection and classification rather than for isolating prognostic methylation patterns independent of disease burden; development of methylation‑based models specifically optimized for prognostic stratification represents an important area for future investigation.

In summary, our findings establish cfMeDIP-seq as a promising and non-invasive approach for the detection of lymphoma. This method could streamline diagnosis, complement existing clinical workflows, and enhance patient care by enabling earlier and more accurate disease characterization.

## Methods

### Patient cohorts

Human subjects research was conducted in accordance with the Declaration of Helsinki. This study was approved by the University Health Network Research Ethics Board (CAPCR 19-5776). We included plasma samples from consenting patients enrolled into an institutional liquid biopsy collection study (LIBERATE, NCT03702309), which specifies the collection of blood samples in Streck tubes, followed by isolation and storage within the University Health Network Biobank, a comprehensive institutional biorepository supporting medical research. We included samples from patients diagnosed with non-Hodgkin lymphoma (DLBCL, FL) or Hodgkin’s Lymphoma (HL). We also included control samples from either patients presenting to our Lymphoma Rapid Diagnosis Clinic and found to have benign conditions, or healthy blood control samples. All participants provided written informed consent for sample collection and research use.

### cfMeDIP-seq and data processing

cfDNA extraction and cfMeDIP-seq were conducted at the Ontario Institute for Cancer Research, as previously described^15^. The resulting libraries were sequenced on Illumina NovaSeq 6000 instruments using 2 × 150 paired end sequencing. Resulting fastq files were used as input for the MEDIPIPE workflow, a comprehensive work-flow for read trimming, alignment, QC, and DNA methylation quantification^28^. Briefly, adapters were trimmed using Trim-Galore^29^. Processed reads were aligned to the human genome (GRCh38) using BWA^30^. The MEDIPS package was used to generate QC metrics and compute raw methylation counts in consecutive 300 bp regions (“windows”)^31^. A strict filtering criterion was set for the number of usable reads, with only those samples having more than 10 million mapped reads retained for downstream analysis. Blacklisted regions were removed, as were genomic regions that are hypermethylated in normal peripheral blood leukocytes, as previously described^32,33^. Regions mapping to sex chromosomes were also removed and only regions with at least 8 CpG sites were retained for downstream analysis. Regions were further filtered to only keep those with at least 5 counts in 10 or more samples. The resulting output was a matrix comprising 212 samples and 30,722 genomic regions. We used trimmed mean of M-values normalization to convert raw methylation counts to log_2_ transformed reads per million. Differential methylation analysis between groups was performed using the limma package (v3.58.1)^34,35^. Regions were considered differentially methylated based on an absolute log_2_ fold-change ≥ 1 and a Benjamini-Hochberg adjusted p-value ≤ 0.05.

### Fragmentomics

The MEDIPIPE workflow utilized Picard’s CollectInsertSizes (v.2.10.9) tool to extract fragment sizes and counts from the BAM files of the 142 discovery Cohort samples. For each sample, fragment counts were normalized and converted to percentages, producing a unique fragment profile. These profiles were combined into a matrix of 142 samples and 900 fragment sizes (1 bp to 900 bp). To focus on the mononucleosome peak, fragments between 30 bp and 250 bp were analyzed using rank 2 non-negative matrix factorization (NMF), which identified two signatures in the data. This analysis was repeated for fragments overlapping hypermethylated regions in lymphoma. Samtools (v.1.20) was used to filter the original BAM files^36^, retaining only fragments that overlapped the hypermethylated genomic regions. The filtered BAM files were then analyzed as described above, generating two new NMF signatures based on the hypermethylated fragment profiles. We complemented the fragment length analysis by calculating sample-specific fragmentation scores^16^.

### Genomic annotation of differentially methylated regions

We used the annotate_regions function in the annotatr package (v1.28.0)^37^ to annotate differentially methylated regions in relation to CpG islands, shores, shelves, and intergenic regions. We used the Genomic Regions Enrichment of Annotations Tool (GREAT, v4.0.4)^38,39^ to describe the distance between differentially methylated regions and TSS. Differentially methylated regions were annotated using annotatePeak from ChIPseeker (v1.38.0)^40^, based on TxDb.Hsapiens.UCSC.hg38.knownGene with TSS defined as ±3 kbp. Gene symbols were retrieved via org.Hs.eg.db, focusing on peaks annotated as “Promoter (≤1 kb)” for downstream analysis. Gene set enrichment analysis was performed using Enrichr^41,42^, focusing on categories related to transcriptional regulation, including ChEA 2022^43^, ENCODE, and ChEA Consensus TFs from ChIP-X^44^, Epigenomics Roadmap HM ChIP-seq^45^, ENCODE TF ChIP-seq 2015, and ENCODE Histone Modifications 2015^46^.

### Targeted capture sequencing and ulpWGS

A subset of samples profiled with cfMeDIP-seq were also subjected to ultra-low-pass whole genome sequencing (ulpWGS) and hybrid capture-based sequencing (capseq), as previously described^47^. Briefly, the ulpWGS genomic libraries were sequenced to a mean coverage target of 0.1 ×, and tumor fraction was computed using ichorCNA^48^. For capseq, libraries were constructed using unique molecular identifiers and a capture panel covering the coding or the hotspot sequences of 80 genes (158 kB). Consensuscruncher was used to collapse reads into single-strand consensus sequences with singleton correction^49^. Mutations were called in tumor-normal mode using Mutect2 (GATK4.1.8.1)^50^. ctDNA concentration was calculated as log haploid genome equivalents per milliliter (log hGE/mL), as previously described by Kurtz et al^12^.

### DNA methylation feature-based classification

Of the 212 samples, 142 were placed into the discovery cohort for machine learning (ML) training and testing. The resulting input was a matrix of 142 samples and 30,723 genomic regions (300 bp). A ML strategy implementing binomial generalized linear models (GLMs) in a one-versus-other classes approach with regularization was adapted from Shen et al^13^. In short, ML training was conducted across 100 iterations, where 80% of discovery cohort samples were randomly selected across each iteration to be included in the training cohort, and the remaining 20% were reserved for inclusion in the testing cohort. Across each iteration, the top 300 differentially methylated regions were selected by limma-trend test-statistic values via a linear model fit for each class compared to other classes. The resulting submatrices of 114 samples and 300 differentially methylated features were used to train a series of one-versus-other regularized binomial GLMs across 100 iterations. GLM training consisted of 10-fold cross validation with 3 repeats across a grid of values for alpha and lambda with optimization for Cohen’s kappa. All resulting models were stored and subjected to testing with each corresponding iteration’s testing cohort (20% of discovery cohort, 28 samples). Following testing, the samples from the validation cohort were classified across all 100 iterations of the trained models, and mean values were collected as a summarized classification score. The performance of classification prediction of the validation cohort was assessed by retrieving the accuracy, sensitivity, and specificity (precision and recall), F1, and receiver operating characteristic (ROC) statistics. This process was repeated for 2-class, 3-class, and 4-class model training.

### Association of methylation scores with clinical parameters

Correlation between methylation score and cell-free DNA concentration was evaluated using Pearson’s correlation coefficient. Comparisons of methylation scores between different disease stages were performed using the Kruskal-Wallis test. For outcome analyses, PFS and OS were estimated using the Kaplan-Meier method, and differences between groups were assessed using the log-rank test.

## Supplementary information

Supplementary information

Supplementary information

## Data Availability

The data associated with this manuscript have been deposited in the European Genome-phenome Archive (accession pending release). The code supporting this study is available at: https://github.com/kridel-lab/cfMeDIP-seq.
