# Supplementary material for: Detection and classification of lymphoma from cell-free methylome data
Source: NPJ Precis Oncol. 2026 Jun 3;10:342. doi: 10.1038/s41698-026-01533-8 (PMC13542107; doi:10.1038/s41698-026-01533-8)
Supplement: Supplementary file 1 [file 41698_2026_1533_MOESM1_ESM.pdf]

Supplementary Data

Supplementary Data 1: Gene Ontology annotation of hypermethylated regions

Supplementary Data 2: Two-class performance metrics

Supplementary Data 3: Three-class performance metrics

Supplementary Data 4: Four-class performance metrics

Supplementary Figure 1

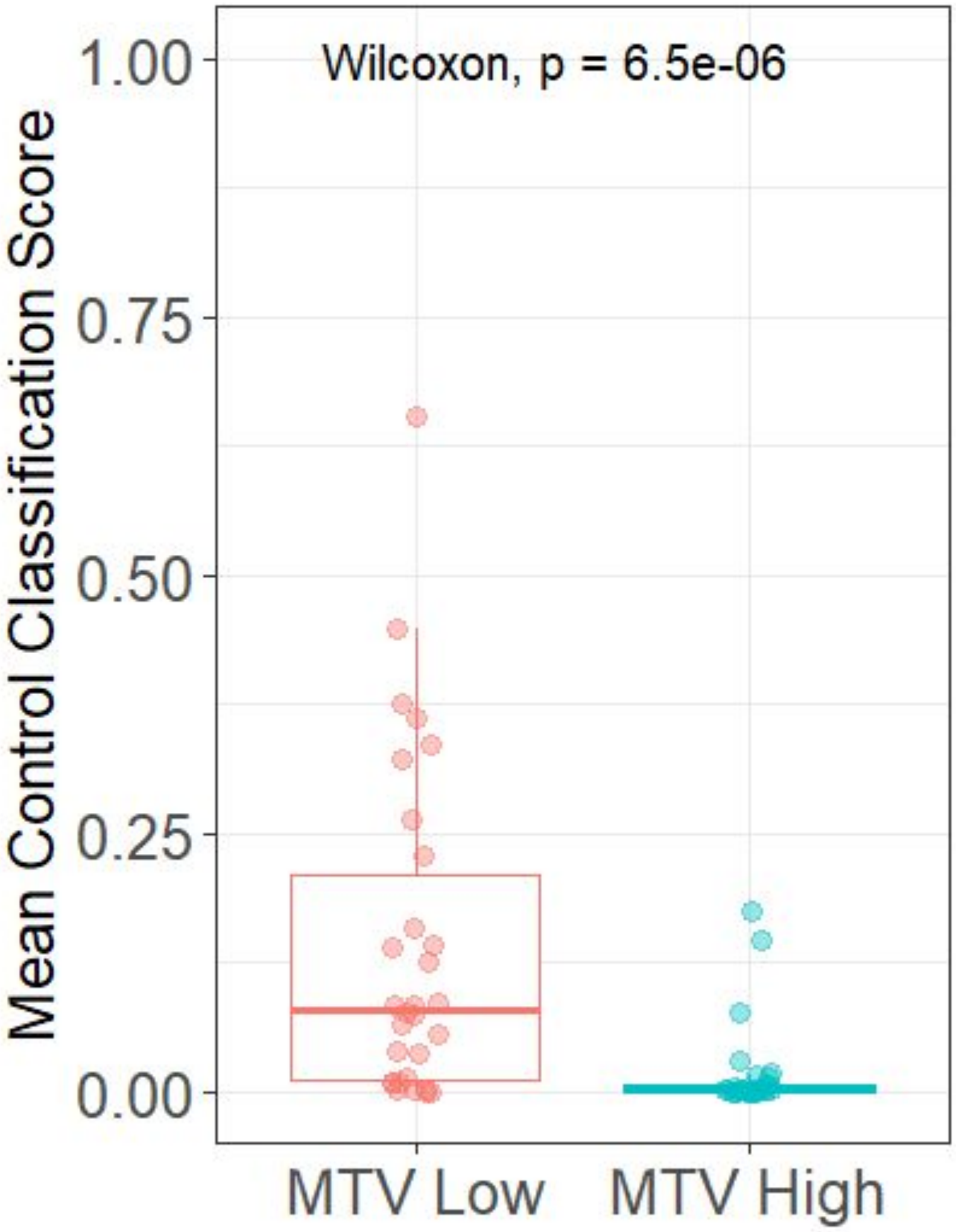

Supplementary Figure 1. Boxplot Showing Mean Control Classification Scores by Metabolic Tumor Volume in Samples from Patients with Diffuse Large B-Cell Lymphoma.

Supplementary Figure 2

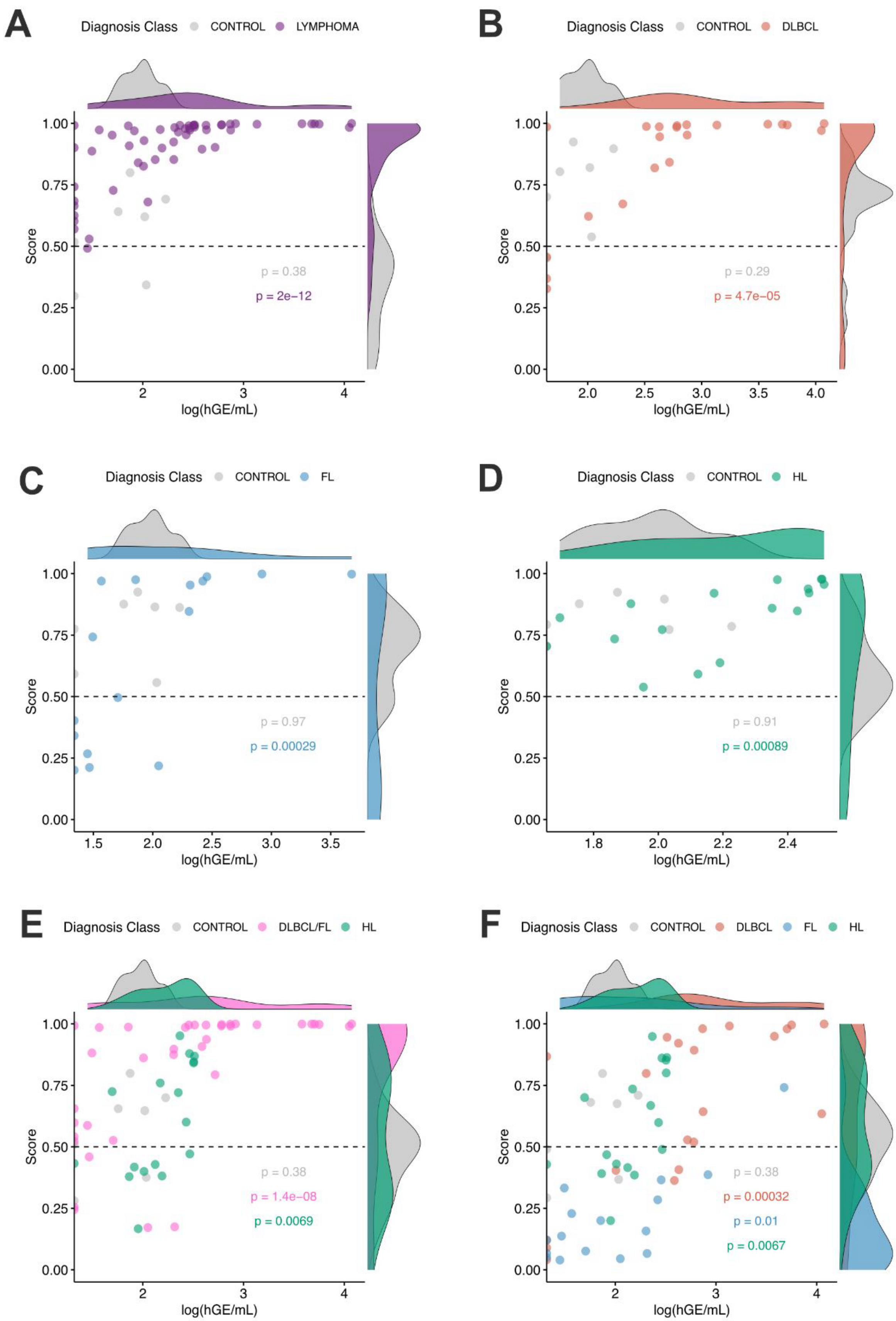

Supplementary Figure 2. Correlation of Classification Area Under the Curve (AUC) Scores with Cell-Free DNA Concentration (log(hGE/mL)). **(A)** Lymphoma–Control. **(B)** DLBCL–Control. **(C)** FL–Control. **(D)** HL–Control. **(E)** HL–DLBCL/FL–Control. **(F)** DLBCL–FL–HL–Control.

Supplementary Figure 3

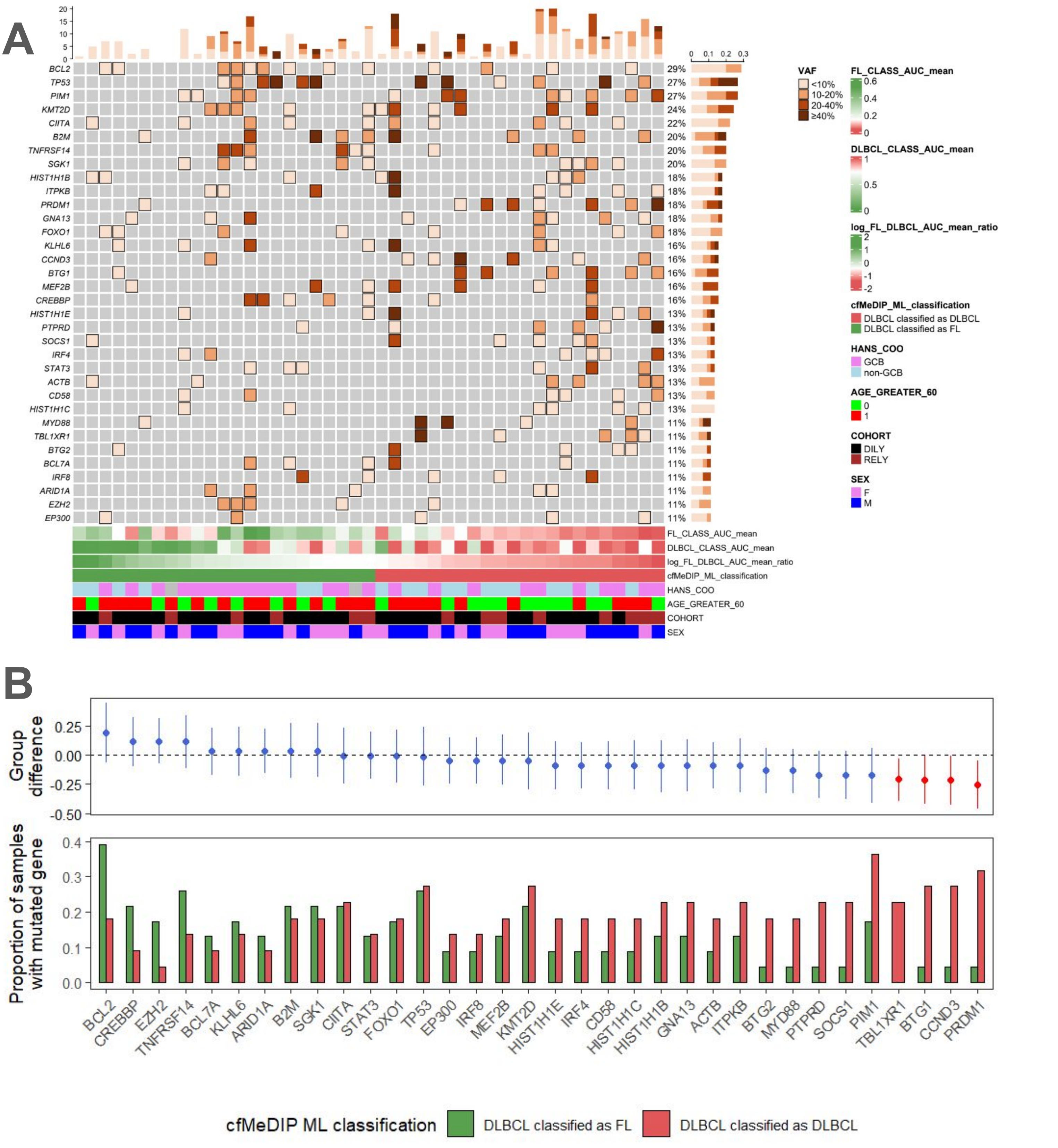

Supplementary Figure 3. Mutation Profiles of DLBCL Samples from Discovery and Validation Cohorts. **(A)** Oncoplot showing the cell-free mutation profiles for each DLBCL sample with matched capseq data. Samples are arranged according to the decreasing log ratio of FL_CLASS_AUC_mean:DLBCL_CLASS_AUC_mean. Recurrently mutated genes (i.e. present in >10% of samples) are shown. **(B)** Group differences for each mutated gene were computed using Bayesian proportion test (https://github.com/rasmusab/bayesian_first_aid). Recurrently mutated genes (i.e. present in >10% of samples) are shown.

Supplementary Figure 4

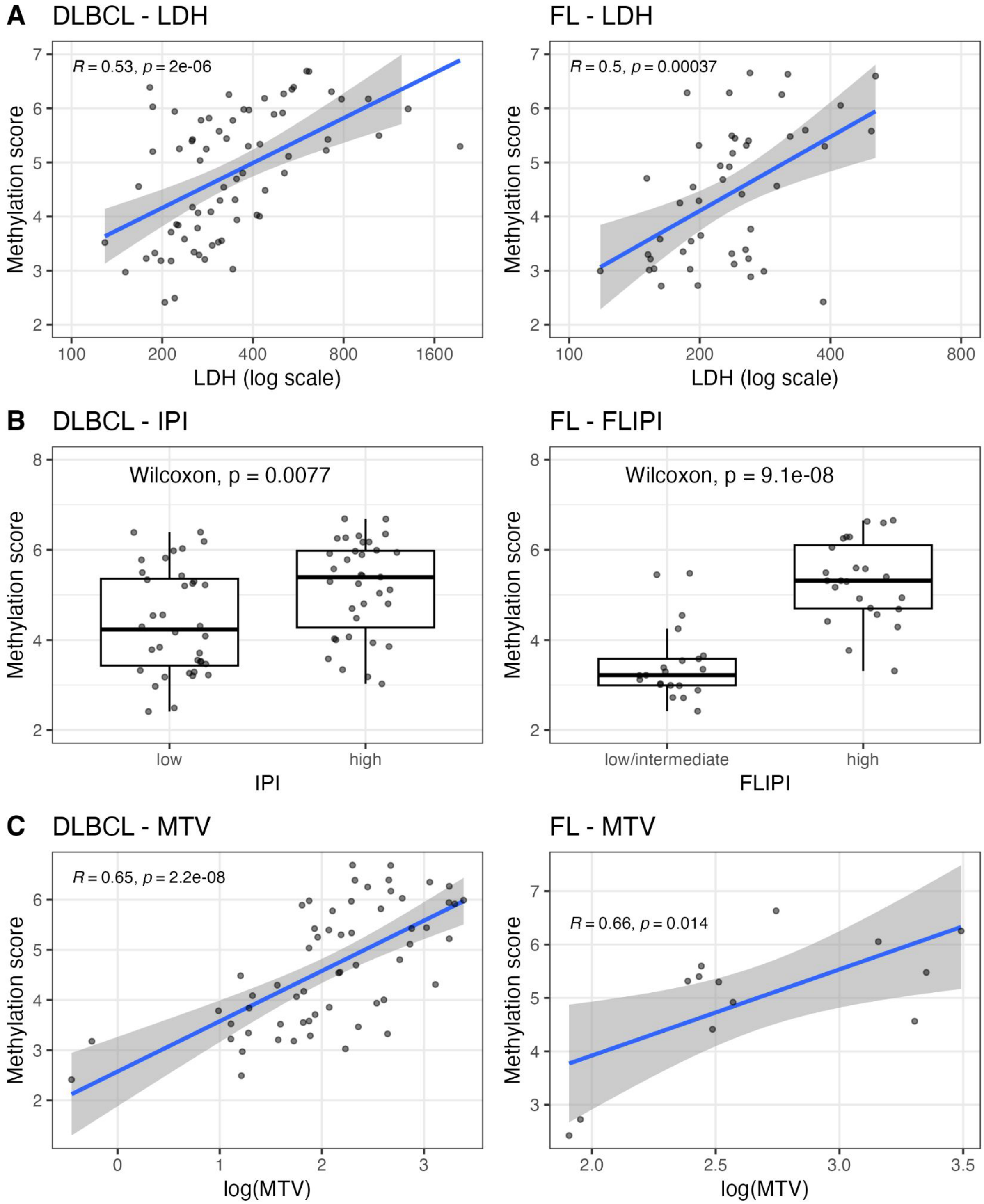

Supplementary Figure 4. Associations Between cfDNA Methylation and Clinical Indices. Scatter plots showing the correlations or associations between cell-free methylation scores and **(A)** serum lactate dehydrogenase (LDH) levels in DLBCL and FL, **(B)** the International Prognostic Index (IPI) in DLBCL and the Follicular Lymphoma International Prognostic Index (FLIPI), and **(C)** metabolic tumor volume.

Supplementary Figure 5

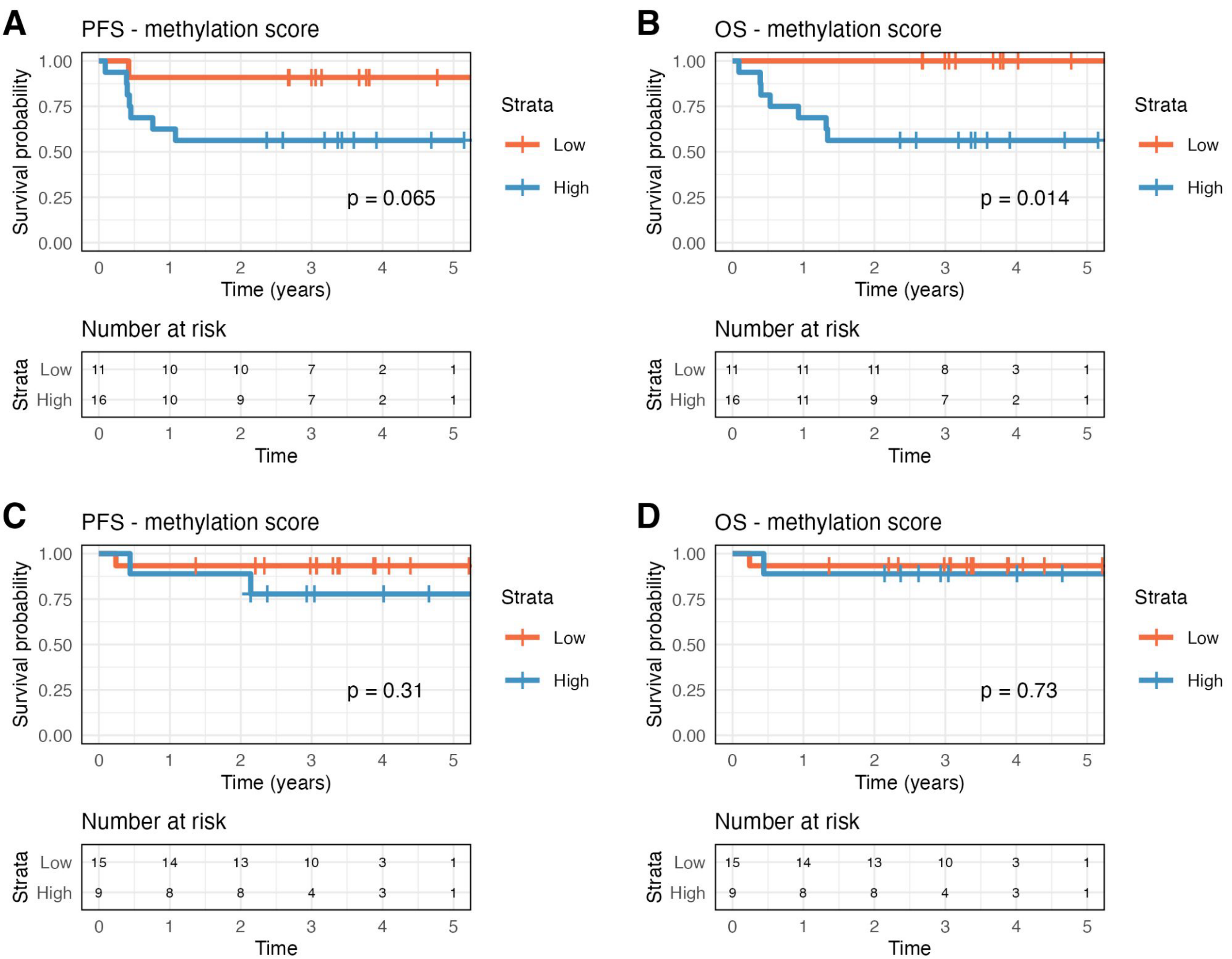

Supplementary Figure 5. Stage-stratified association between cell-free methylation scores and clinical outcomes in DLBCL. **(A, B)** Kaplan–Meier curves showing progression-free survival (PFS, left) and overall survival (OS, right) among patients with advanced-stage (Ann Arbor III–IV) DLBCL, stratified by cfDNA methylation score (high vs. low, dichotomized at the cohort median). **(C, D)** Kaplan–Meier curves showing PFS (left) and OS (right) among patients with limited-stage (Ann Arbor I–II) DLBCL, stratified by cfDNA methylation score using the same cutoff. Log-rank p-values are shown.
